# Supplementary figures and images for: Further characterization of the effect of the prototypical antidepressant imipramine on the microstructure of licking for sucrose
Source: PLoS One. 2021 Jan 15;16(1):e0245559. doi: 10.1371/journal.pone.0245559 (PMC7810301; doi:10.1371/journal.pone.0245559)

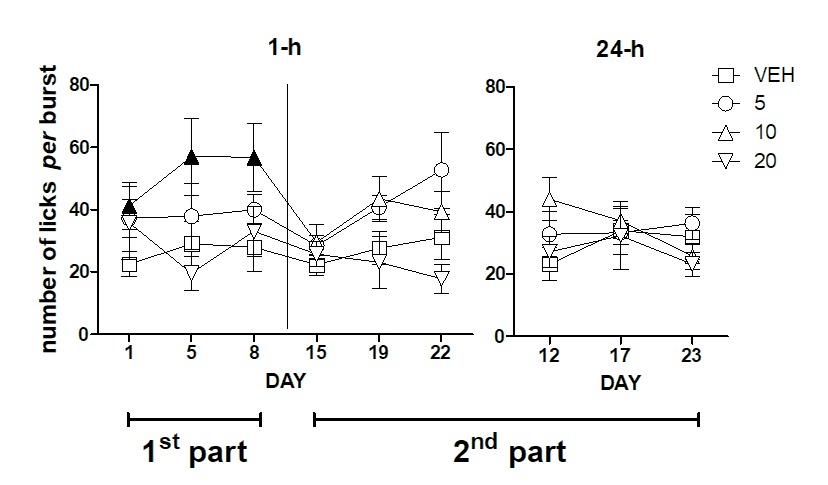

Supplement: S1 Fig — (TIF) [file pone.0245559.s002.tif]

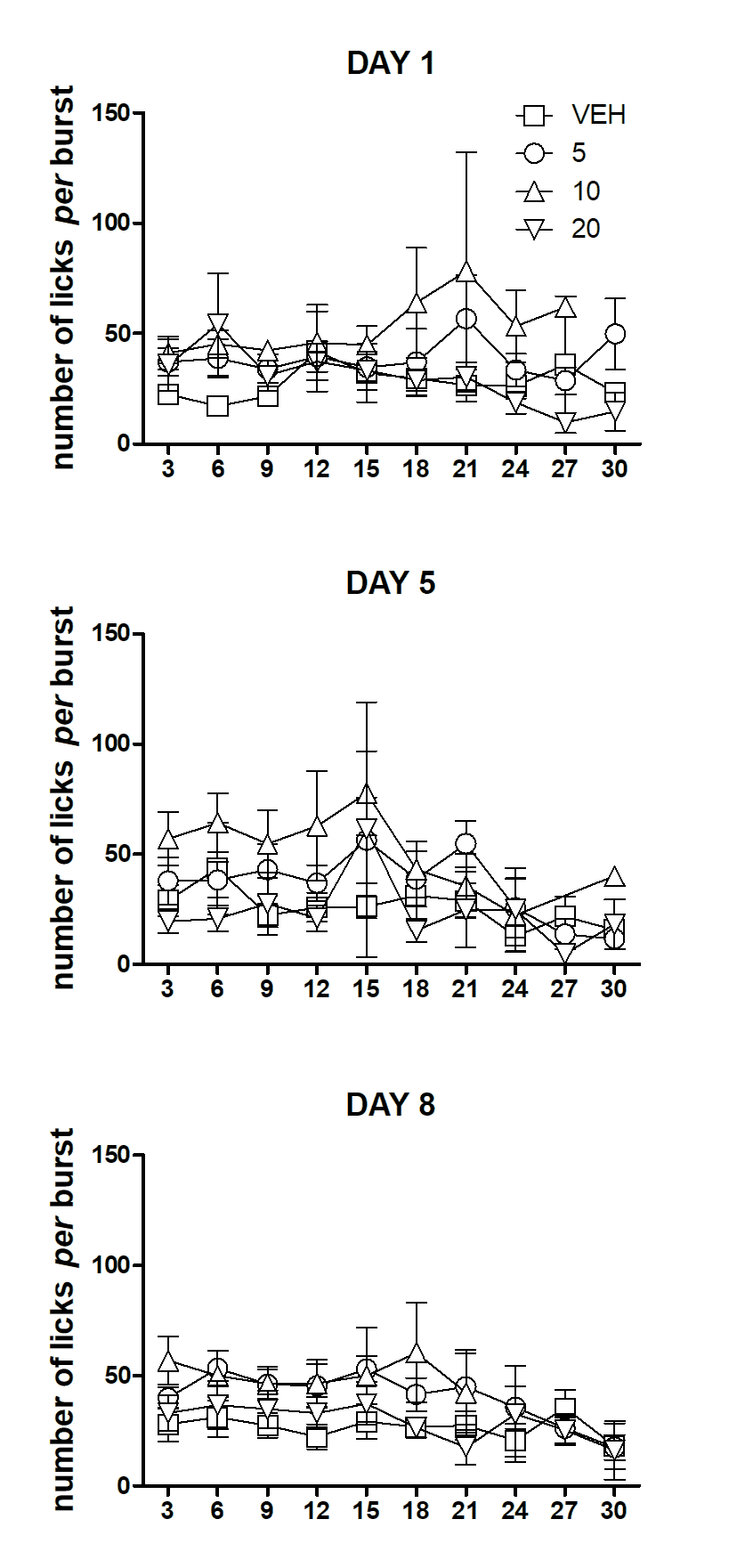

Supplement: S2 Fig — (TIF) [file pone.0245559.s003.tif]
