# Supplementary material for: Further characterization of the effect of the prototypical antidepressant imipramine on the microstructure of licking for sucrose
Source: PLoS One. 2021 Jan 15;16(1):e0245559. doi: 10.1371/journal.pone.0245559 (PMC7810301; doi:10.1371/journal.pone.0245559)
Supplement: Attachment — Submitted filename: Response to Reviewers.doc [file pone.0245559.s005.doc]

**Response to Reviewers**

The text by the Reviewer is in Italics. Please notice that in the Revised Manuscript with Track Changes the new text is highlighted with a light grey background. Numbers in square brackets refer to citations included in the References of the submitted paper.

*Reviewer #2: The paper is greatly improved by reframing of it in terms of examining the “immediate” effects of the imipramine on sucrose licking patterns in the absence of interposed post-session administrations, as opposed to invoking some examination of learning or memory. In general, the discussion is a lot more cautious about mechanism, which is appropriate given the design and data. I still have a couple of outstanding concerns.*

Response: We thank the Reviewer for this comment.

*Perhaps the authors could add some indication of which sessions are included in part 1 and 2 in the table. But, in general, the table helps clarify the protocol.*

Response: Table 1 was integrated with the requested indications (clean ms: page 4; rev. man. with track changes: page 5).

*I would like to see more of the within session data (plotted across time). Per my previous review, I thought it would be important to examine burst size in addition to be able to interpret the burst number data across the session. The authors now present the first 3 min burst size in the supporting information, but not beyond that. The authors appear to favor the interpretation that imipramine reduces behavioral motivation or increases satiation. But for the 10 mg dose (and maybe the 5), the data could just as easily be interpreted as increase in “hedonic value” based on the framework provided by the authors in the introduction. In fact, the first 3 minute burst data, which overlap well with the overall session burst data, in Fig S1, would seem to indicate that burst size is increased prior to satiation signals coming online (i.e., in the early part of the session). Perhaps looking at dynamic changes in lick rate (or burst size) in the 3 min bins across the entire session would clarify. The burst size does not appear to be reduced until the second phase of the experiment. This may be a limitation of interpreting burst size as a function of reward and burst number as a function of motivation. Of course, it is impossible to know whether this is simply due to an accumulation of drug or something about the pre and post injection procedure. I suppose, at the very least, if the drug at these doses was increasing the rewarding value of sucrose, it did not drive overconsumption (intake was pretty stable across the days).*

Response:

1) The within-session time-course of burst size of the first three sessions (i.e. of the sessions for which it is reported the within-session time-course of burst number, as requested by the Reviewer in the previous run of this reviewing process) was provided as Supporting information (S2 Fig, see also S1 Data for the individual numbers). These data do not seem to show changes in burst size in the course of the session. However, an important limitation to the possibility to make a meaningful interpretation of these data comes from the fact that, as the sessions proceeds, the subjects stop licking, thus resulting in empty cells late in the session. Firstly, this leads to an exclusion of too many subjects from the repeated measures ANOVA (which does not allow empty cells). Maybe more importantly, the lack of a relevant number of subjects in the late session time bins – which are not necessarily the same in different time bins – brings as a consequence that the comparisons between the different time bin values early and late in the session are heavily affected by differences between subjects (which are inextricably confounded with possible changes in time within the session). The data relative to the within-session time course of burst size are described in the Results section (clean ms: lines 237-244; rev. man. with track changes: lines 244-251) and mentioned in the Discussion (clean ms: 331-332; rev. man. with track changes: lines 339-340). We wish to stress that this experiment was designed in order to consider the whole session burst size as the relevant measure, as highlighted in the Discussion (clean ms: 385-397; rev. man. with track changes: 394-406). Indeed, the best way to investigate changes of burst size in the course of ingestion involves the recording of the data in relation to the progression of a meal, rather than in relation of a fixed time session (e.g. [10]).

2) We absolutely agree with the Reviewer's considerations in regard of the interpretative issues. As acknowledged by the Reviewer in the preliminary remark, in general, the previously submitted text (i.e. the first revision) is a lot more cautious in the formulation of the proposed interpretations. In particular, following this Reviewer's advice, in the first revision we acknowledged the limitations in regard of the interpretation of the differences between the first and the second part of the experiment. Moreover, all the statements about the interpretation of the results in terms of reduced motivation were formulated in cautious terms (e.g. “suggest”: clean ms: 325, 400; rev. man. with track changes: line 333, 409; “possibly due”: clean ms: 420; rev. man. with track changes: 429). However, although the possibility that the increased burst size might be due to an increased hedonic impact was not dismissed in the previous version (please see final part of the conclusive remarks of the discussion, unmodified), we must admit that the formulation of the text “downplayed” this interpretation. Thus, in the currently submitted text we made these changes/integrations:

-“is not” was changed into “does not appear to be” (clean ms: 378-379; rev. man. with track changes: 386-387).

- It was added the following sentence: “Nonetheless, further experiments are necessary to determine whether the effect of imipramine on burst size might be accounted for by an increased hedonic impact.” (clean ms: 397-398; rev. man. with track changes: 406-407).

*Line 45-46 is a little unclear. Burst number cannot be separated from orosensory cues in this design.*

Response: We agree with the reviewer. Indeed, one of the central interests in our previous work on licking is about the interpretation of the within-session changes of burst number in response to changes in sucrose concentration or to treatments which might affect the hedonic response to taste (e.g. [4,22,26,39]). To clarify the point indicated by the Reviewer in the Introduction, with added a note in parentheses (clean ms: lines 49-50; rev. man. with track changes: lines 52-53). This required a rearrangement of the sentence order within the paragraph (rev. man. with track changes: lines 43-54). In particular, we left unchanged the statement “The number of licking bursts […] is particularly sensitive to stimuli […] which do not involve the orosensory contact with the reward.”, and added a cautionary note: “(But notice that this measure does respond to orosensory cues, albeit in a less straightforward fashion than burst size.)”.

***

Finally, we wish to thank both the Editor and the Reviewers for their helpful and constructive comments.

***
